# Characterization of a circulating PRRSV strain by means of random PCR cloning and full genome sequencing

**DOI:** 10.1186/1743-422X-8-160

**Published:** 2011-04-10

**Authors:** Jan Van Doorsselaere, Marc Geldhof, Hans J Nauwynck, Peter L Delputte

**Affiliations:** 1 Department of Health Care and Biotechnology, KATHO Catholic University College of South-West Flanders, Wilgenstraat 32, 8800 Roeselare, Belgium; 2 Department Virology, Parasitology and Immunology, Faculty of Veterinary Medicine, Ghent University, Salisburylaan 133, 9820 Merelbeke, Belgium; 3 ProVaxs, Faculty of Medicine and Health, Ghent University, De Pintelaan 185 - 3K3, 9000 Gent, Belgium

## Abstract

PRRS is a pig disease of major economic importance that causes respiratory and reproductive problems in pigs. Over the last years it has become clear that PRRSV heterogeneity is increasing. Consequently, this has a potential impact on diagnosis and strategies to counter this disease. The use of sequence-independent PCR techniques for the detection and characterization of PRRSV could be useful to bypass problems associated with the heterogeneity of this virus.

A random PCR cloning approach was tested for the characterization of PRRSV strain 07V063 of unknown genetic background that circulated on a Belgian farm. By using this approach, 7305 bp of sequence data were obtained, distributed randomly across the genome. Using RT-PCR with strain-specific primers, the full length sequence (15014 nt) was obtained. Phylogenetic relationships using ORF5 and ORF1a (NSP2) sequences showed that 07V063 was classified in type 1 subtype 1 and that 07V063 was genetically different from prototype Lelystad Virus (LV). 07V063 showed 87-93% aa identity with LV ORFs coding for structural proteins. Most variation (compared to LV) was noticed in Nsp2 (81% identity) with a deletion of 28 aa. This deletion was different from other known deletions in this ORF. In conclusion, it is shown that this random PCR cloning approach can be used for the characterization of new PRRSV strains of unknown genetic background.

## Findings

Porcine reproductive and respiratory syndrome (PRRS) is an economically important viral pig disease in swine producing countries worldwide. The virus can cause reproductive disorders and can give rise to respiratory problems in pigs of all ages [1]. Prevention of the disease is based on a combination of management and vaccination. Evidence is accumulating that PRRSV heterogeneity is affecting the vaccination efficiency. It is suggested that vaccines are only efficacious when the vaccine virus and the challenge virus share a sufficiently high homology [2-6]. PRRSV heterogeneity was originally considered mainly to occur between European (genotype 1) and American type (genotype 2) PRRSV, but current understanding shows a more complex situation with considerable genetic variability within genotypes [7-9]. Since such variability may affect the efficacy of vaccination programs and pose an obstacle for PRRSV prevention and control, knowledge on the PRRSV strains circulating on a farm may be essential for choosing an appropriate vaccine [10].

PRRSV diagnosis is mainly based on detection of PRRSV antibodies, Reverse Transcriptase (RT) PCR or virus isolation. Detection of antibodies by ELISA or IPMA is not sufficient to establish the level of PRRSV heterogeneity [11]. RT-PCR allows rapid detection and genotyping of PRRSV, but the high degree of sequence variation observed for PRRSV can influence results obtained by (real-time) RT-PCR and primers and/or probes should be carefully designed based on conserved regions [8,12]. The development of sequence-independent PCR techniques could be useful for the diagnosis and genotyping of unknown PRRSV isolates and for assessment of the PRRSV heterogeneity of field isolates. Several methods have been developed for the identification of viruses without prior sequence knowledge [13]. For instance, whole genome amplification and random PCR are relatively simple. In both these methods, viral particles (from biological samples or cell culture) are treated with DNAse and RNAse to remove contaminating nucleic acids. RNA and/or DNA from the viral particles is extracted and RNA is reverse transcribed to cDNA using a primer with a random 3'end. Subsequently, cDNA or viral DNA is amplified using a shorter primer (without the 3' random end). This results in DNA fragments of varying size (e.g. 0.5 - 2 Kb) and these fragments can be cloned and sequenced. For instance Allander *et al. *[14] used random PCR on human respiratory tract samples which allowed identification of several unknown viruses.

The aim of this study was to test a random PCR cloning technique [14] for the detection and genotyping of a PRRSV strain of unknown genetic background.

### Random PCR cloning for the identification of PRRSV 07V063

**Figure 1:**
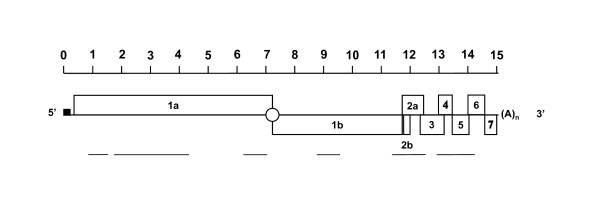
**PRRSV genome and position of the contigs**. The sequences obtained in the random PCR cloning approach were assembled in six contigs (with sizes between 622 and 2072 bp) dispersed over the genome. The contigs are shown with a line.

PRRSV 07V063 was isolated from an aborted foetus from a Belgian farm, by inoculation of porcine alveolar macrophages. On this farm, vaccination with Porcilis™ was in place. PRRS diagnosis was confirmed upon detection of cytopathic effect (CPE), and detection of PRRSV antigens by IPMA staining with the nucleocapsid specific mAb P3/27 [15]. The use of a random PCR approach abrogates the need for *a priori *sequence information and in combination with small scale shotgun sequencing, this can result in viral sequences. Virus 07V063 was grown on MARC-145 cells and concentrated as described [16] and the viral pellet was treated with DNAseI and RNAse. RNA was extracted using commercial kits and used in reverse transcription and random amplification using the tagged random hexanucleotide 5'-GCCGGAGCTCTGCAGATATCNNNNNN-3' for both first- and second strand cDNA synthesis and subsequent amplification of the cDNA with primer 5'-GCCGGAGCTCTGCAGATATC-3' [14]. Random PCR fragments ranging between 500 and 1200 bp were cloned in pCR-Blunt II-TOPO (Invitrogen). Twenty nine clones were sequenced as described [17]. Twenty three clones (80% of the clones) contained PRRS sequences (Table 1). The six other clones showed no match when performing BlastN http://www.ncbi.nlm.nih.gov. The 07V063 sequences were randomly distributed across the PRRSV genome. Several clones were overlapping and six contigs (with sizes between 622 and 2072 bp) were obtained (Figure 1). Thus, without prior knowledge of the sequence it was possible to obtain 7305 bp sequence data using a random PCR cloning approach, hereby confirming PRRS identity.

**Table 1:** Overview of the sequences from 07V063 obtained by random PCR cloning.

Clone	Size (nt)	Position	% nt identity
49	671	774-1444	81
73	198	1692-1889	89
104	826	1808-2633	89
20	798	2616-3413	86
105	375	3069-3443	88
88	429	3420-3847	93
33	332	3957-4288	91
92	316	6198-6512	93
61	713	6367-7079	93
103	312	6768-7079	94
35	364	6500-6863	93
12	247	8132-8378	89
51	627	8931-9557	86
80	358	9200-9557	87
82	622	11225-11846	87
11	258	11225-11482	86
81	601	11928-12528	92
70	189	12336-12524	94
40	277	12364-12640	90
78	395	12991-13385	90
57	935	13195-14129	91

The position of the sequences is indicated relative to LV. % nt identity is with LV.

### Full length sequence of 07V063 and comparison with prototype LV

To allow a more detailed evaluation of the PRRSV isolate 07V063, the full length genome sequence was obtained using primers that were based on the 07V063 sequences from the random PCR cloning approach (Table 2). Overlapping amplicons (spanning the complete genome) were obtained using RT-PCR. Both strands of these fragments were directly sequenced. For the amplification of the 3'end, oligodT was used in combination with ORF7fw. A 5' end primer (5'endfw) was designed based on the alignment of genotype 1 strains LV (M96262), EuroPRRS (AY366525), SD01-08 (DQ489311), KNU-07 (FJ349261) and HKEU16 (EU076704). This primer was used in combination with primer Lavgsprev to amplify the 5'end. A full length sequence of 15014 nt was obtained. This sequence was deposited in Genbank (Accession GU737264).

**Table 2:** Oligonucleotide primers used in RT-PCR amplification and nucleotide sequencing of 07V063

Primer	Sequence	Position
5'endFW	atgatgtgtagggtattccccc	1-22
Orf1univFW	ccctttaaccatgtctggc	111-130
Orf1-1fw	catcc gggtg ctgctgg ctt	336-355
Orf1-2fw	ggag ccaccc acgtgtt gac	681-701
Lav49fw	aatcaatggtattcgtgctg	1072-1091
Orf1-3-fw	tcaat gcctacaa ctgcccg	1631-1650
Orf1-4-fw	cttgta taaa ttgct attgg	1988-2007
Orf1-5-fw	acaa cagg cctc gtaa ggg	2472-2490
Lav73fw	aaaacttggcgctgcacgtc	3102-3121
Orf1-6fw	ggtcc atta gcca gcgcct	3451-3469
Orf1-7fw	cttgag cagcg ccaa cattg	3686-3705
Lav33fw	ggtgttggcacggcgagag	4129-4147
Orf1-8fw	catgg ctgtt gccca agtgt	4538-4557
Orf1-9fw	ttgt gctt acgcc tggccca	4859-4878
Orf1-10fw	ggcgac tcct ataat cgtat	5364-5383
Orf1-11fw	ccaa gcac ttcg cagg tccg	5701-5720
Orf1-12fw	ggctt ggctg ccgaaa tcgg	6096-6115
Orf1-13fw	aatgaa gggag tctt gtcta	6566-6586
Lav92fw	gtgtatccctcggctaccac	6891-6911
Orf1-14fw	catta gtcaa cttcaa ggtt	7280-7299
Orf1-15fw	gga ccc tga gcgg catgaa	7765-7783
Lav12fw	ccaagaactccatggcaggt	8172-8191
Orf1-16fw	ggaaaaacaaattcaaggag	8442-8461
Orf1-17fw	tccag cccatg ctggt ata	8817-8835
Lav51fw	gtgtttgtttcactcacact	9316-9335
Amp6fwint	catcagaccatgtttgacat	9764-9783
Orf1-18fw	aaggc caggaa cacca gggt	10136-10155
Orf1-19fw	cccagta tttgca ccttt gc	10633-10652
Orf1-20fw	cggccgta cttgc aaccag	11132-11150
Orf2afw	gts aca cck tat gatta cg	11387-11406
LavORF2aseqfw	gtgttcgacaacgcccacacgc	11577-11598
Orf3fw	agcc taca gta caa ca ccac	12234-12253
LavORF3seq1fw	agcgttgagctcatcttccc	12261-12280
Orf4fw	cgg ccc ait tcc atccigag	12672-12691
Orf5Pesfw	tga tca cat tcg gtt gct	13320-13337
Orf6fw	tacc aa ctt tc ttc tggac	13838-13856
Orf7fw	tgg cccc tgccc aic acg	14328-14345
Orf1-1-rev	gtcaa cacgt gggtgg ctcc	701-681
Lavgsprev	cgacttgacattctagtcca	900-881
Orf1-2-rev	agat gcca aacgg acgaa cc	1304-1285
Orf1-3-rev	gcag cctt cgga gcag acgc	1796-1777
ORF1-4-rev	cggtg aaca cgag acacc tg	2252-2233
Orf1-5-rev	gctg atgt tgtc ggatt ctg	2615-2596
Orf1-6-rev	ctggg aaca ggagg cgg tgt	3202-3182
Orf1-7-rev	gggttgg atg gagtc gagaa	3730-3711
Lav33rev	ccccaacacttgtgacaacg	3982-3963
Orf1-8rev	gt ccgag tccac tacaatc	4403-4385
Orf1-9rev	agag ttgt gccac tgct gaaa	4755-4735
Amp3intrev2	cagagaaggccggttattcct	5023-5003
Amp3intrev	gattccaatgagatcacca	5609-5591
Orf1-10rev	gctc ggac taaaa cagc tgg	5959-5940
Lav92rev	caccaatgatgatgataggg	6222-6203
Orf1-11rev	cttg caca gaca cagtttt	6720-6702
Orf1-12rev	ttcaa ggca gttg tca ggct	7190-7171
Orf1-13rev	tca ttaa gacg acacc ggaa	7406-7386
Orf1-14rev	cttg ccat cgga cacaa gg	7903-7885
Orf1-15rev	tga cacc actg agcg ccga	8396-8378
Orf1-16rev	agaca cact ggtg acggggt	8696-8676
Lav51rev	aagaaagctgggtttgtcag	8971-8952
Orf1-17rev	cggaa tctg tttcaa cacag	9460-9441
Orf1-18rev	ccagg tggtt gcaa tatcca	9944-9925
Orf1-19rev	aaaactccc gaag ttggtcg	10385-10366
Orf1-20rev	aggc ttgc tgtag tgggcat	10762-10743
Lav82rev	ttcaagctggaagtaggc	11244-11225
Orf1-21rev	tgatttt gctcc acag tgac	11741-11722
Orf2arev	tcatr ccc tatt y tgc acca	12558-12539
Orf3rev	agaa aa gg cacgc ag aaa gca	13184-13165
Orf4rev	cattcagctcgcataicgtcaag	13569-13547
Orf5Pesrev	ggg cgt ata tca tta tag gtg	14100-14079
Orf6rev	acccagc aa ctgg cacag	14606-14589
Orf7rev	tcg ccc taa ttg aa tagg tga	14966-14946

**Figure 2:**
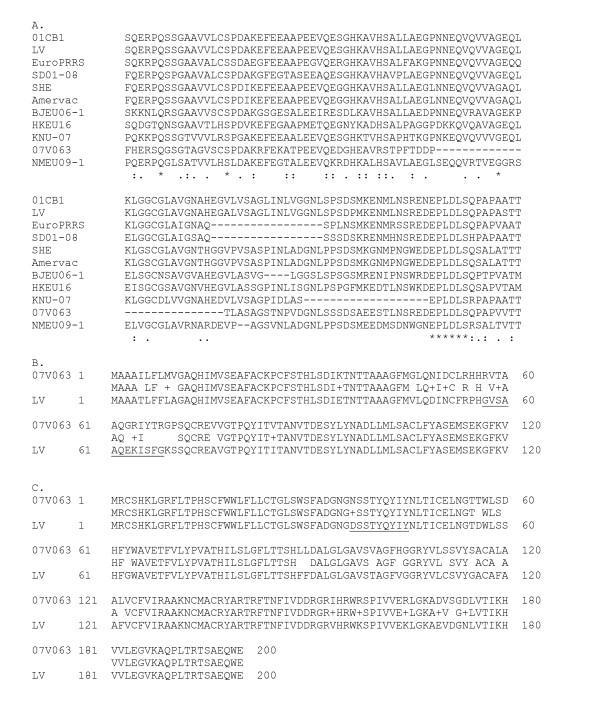
**Alignment of Nsp2, ORF4 and ORF5 proteins from 07V063 with LV (ORF4 and ORF5) and a selection of genotype 1 strains (Nsp2)**. A. Alignment of Nsp2 proteins from genotype 1 strains. Only aa positions 636-755 (LV) are shown. The deletion in 07V063 is located at aa positions 683-710. B. Alignment of GP4 from 07V063 and LV (only the first 120 aa are shown). A neutralizing epitope in LV (57-68) is underlined. C. Alignment of GP5 from 07V063 and LV. A neutralizing epitope in North American strains (37-45) is underlined.

The 5' end and 3'end of 07V063 was 221 nt and 114 nt, respectively. The size of the 5'end of 07V063 is identical with the 5'end of LV with 92.3% identity and 17 nt differences. Several motifs such as the transcription regulatory sequence (UUAACC) and CACCC stretches (involved in binding of host cell transcription factors) are conserved in 07V063 [18]. Table 3 gives an overview of all ORFs in the 07V063 genome and comparison with ORFs from prototype LV. Most variation with LV was noticed in Nsp1 (85% identity/91% similarity) and Nsp2 (81% identity/85% similarity). A major difference is a deletion of 28 aa in a variable region of Nsp2 (at positions 683-710). Similar deletions in this region are known e.g. EuroPRRS has a 17 aa deletion (Figure 2A; [18]). The deletion in NSP2 in 07V63 could be a unique marker for this strain.

**Table 3:** Comparison of proteins from 07V63 and prototype LV

*ORF*	*Protein*	*Size 07V63*	*Size LV*	*% identity*	*% similarity*
*1a*	Nsp1	*385*	*385*	*85*	*91*
	Nsp2	833	861	81	85
	Nsp3	447	447	93	96
	Nsp4	203	203	92	96
	Nsp5	170	170	96	97
	Nsp6	16	16	100	100
	Nsp7	269	269	96	97
	Nsp8	45	45	100	100
1b	Nsp9	645	645	96	98
	Nsp10	442	442	94	97
	Nsp11	224	224	95	97
	Nsp12	152	152	93	96
2a	GP2	249	249	93	94
2b	E	70	70	95	97
3	GP3	265	265	89	92
4	GP4	183	183	87	93
5	GP5	200	201	91	94
6	M	173	173	93	94
7	N	128	128	91	98

Strain 07V063 showed 87 - 95% aa identity with LV for the structural ORFs 2 - 7. We compared GP4 and GP5 proteins from 07V063 and LV since it has been shown that these proteins are the main target for neutralizing antibodies. Figure 2B shows an alignment of ORF4 proteins. Notably is the high variation in the region 50-70. It has been shown that a neutralizing epitope is present in LV at positions 57-68 [19] and that this region is under antibody-mediated pressure *in vitro *and *in vivo *[20,21]. Pigs infected with 07V063 produce neutralizing antibodies against the ^57^RVTAAQGRIYTR^68 ^epitope. However, these antibodies do not cross-protect against LV [22]. Similarly, antibodies against the same region in LV, do not cross-protect against 07V063. Interestingly, this lack of cross-neutralization is in agreement with the finding that strain 07V063 was able to replicate and cause disease on a farm where animals were vaccinated with the LV-like Porcilis™ vaccine.

GP5 has been described as the main target for virus-neutralizing antibodies in North American PRRSV strains. A neutralizing epitope has been identified at positions 37-45 [23]. Figure 2C shows that 07V063 and LV have an identical sequence from 37-45 with the exception of an extra glycosylation site at position 37 in 07V063. It has been shown that several strains are glycosylated at this position but the significance of this glycosylation is not known. Other amino acid changes occur throughout the sequence and several of these positions have been described as variable [24]. No other differences in glycosylation pattern of the structural proteins between 07V063 and LV was observed.

### Phylogenetic relationship of 07V063

Since ORF5 is frequently used as a marker for the study of genetic relationships [8], we constructed phylogenetic trees using ORF5 sequences from a selection of genotype 1 strains (Table 4). In addition genotype 1 strains for which the full length sequence was available in Genbank were included. VR-2332 (genotype 2) was used as out-group.

**Table 4:** Overview of strains used for phylogenetic analysis

Strain	Genotype	Genbank Accession ORF5	Genbank Accession ORF1a (nsp2)
VR-2332	2	U87392	U87392
Lelystad	1 (subtype 1)	M96262	M96262
EuroPRRS	1	AY366525	AY366525
01-CB1	1 (subtype 1)	DQ864705	DQ864705
Amervac	1 (subtype 1)	GU067771	GU067771
HKEU16	1 (subtype 1)	EU076704	EU076704
KNU-07	1 (subtype 1)	FJ349261	FJ349261
SHE	1 (subtype 1)	GQ461593	GQ461593
SD01-08	1 (subtype 1)	DQ489311	DQ489311
BJEU06-1	1 (subtype 1)	GU047344	GU047344
NMEU09-1	1 (subtype 1)	GU047345	GU047345
07V063	1 (subtype 1)	GU737264	GU737264
			
PyrsVac	1 (subtype 1)	DQ324681	ND
Porcilis	1 (subtype 1)	AAW78901	ND
Olot/91	1 (subtype 1)	X92942	ND
Yuz-34	1 (subtype 3)	DQ324692	ND
Bel-42	1 (subtype 3)	DQ324669	ND
Obu-1	1 (subtype 3)	DQ324671	ND
Soz-6	1 (subtype 3)	DQ324686	ND
Dzi-62	1 (subtype 1)	DQ324675	ND
Cresa11	1 (subtype 1)	DQ009626	ND
IV3140	1 (subtype 1)	DQ355821	ND
28639/98	1 (subtype 1)	AY035912	ND
361-4	1 (subtype 1)	AY035915	ND
Sno-4	2 (subtype 2)	DQ324683	ND
Sid	2 (subtype 2)	DQ324682	ND
Aus	2 (subtype 2)	DQ324667	ND
Okt-35	1	DQ324677	ND
16/2000	1	DQ345743	ND
SD02-11	1 (subtype 1)	AY395078	AY383634
SD01-07	1 (subtype 1)	AY395079	AY383632
SD03-12	1 (subtype 1)	AY395074	AY383635
SD03-15	1 (subtype 1)	AY395076	AY383636
It-22	1 (subtype 1)	AY739978	ND
It-39	1 (subtype 1)	AY739995	ND
It-44	1 (subtype 1)	AY740000	ND
It-35	1 (subtype 1)	AY739991	ND
It-13	1 (subtype 1)	AY739969	ND
Lena	1 (subtype 3)	EU909691	ND

The type of the strains is according to Stadejek et al (2008). ND = no data. VR-2332 is genotype 2. Eleven genotype 1 isolates for which full length sequences were obtained are listed first.

**Figure 3:**
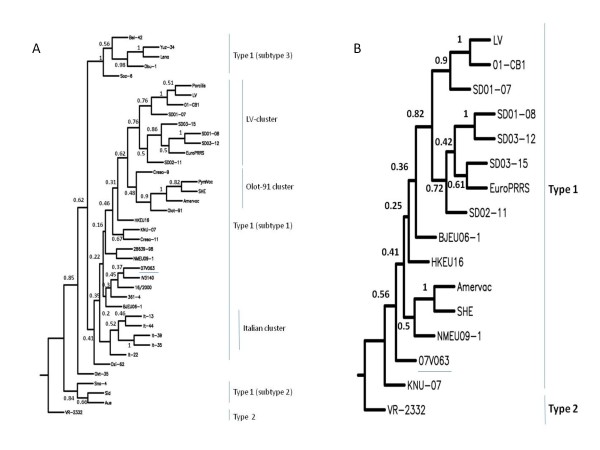
**Phylogenetic relationship of 07V063**. Phylogenetic trees were derived from multiple sequence alignments using Phylip v3.67. Bootstrapping was performed 500 times using SEQBOOT. Pairwise distances between DNA and/or protein sequences were determined with DNAdist and PROTdist, respectively. Neighbour-Joining (NJ) trees were calculated with NEIGHBOUR and Maximum Likelyhood (ML) trees with DNAML and PROML. Majority rule consensus trees were calculated using CONSENSE. The percentage confidence is indicated on the branches (500 datasets). Trees, constructed using NJ method, based on ORF5 DNA (A) or ORF1a (Nsp2) DNA (B) sequences. Strain 07V063 is underlined. VR-2332 was used as outgroup.

Figure 3A shows a phylogenetic tree of ORF5 DNA sequences based on the Neighbour Joining (NJ) method. Several clusters are evident and supported by high bootstrap values. It can be concluded that 07V063 clusters within the pan-European subtype 1 [8]. Within subtype 1, a cluster with LV- and Olot/91-like strains can be distinguished. Although both LV and Olot/91 belong to the earliest PRRSV isolates, still LV and Olot/91-like strains such as SD01-08 are circulating. Strain 07V063 is genetically different from LV- and Olot/91- like strains. Apparently 07V063 clusters together with isolates from different geographical locations e.g. isolates from Spain (16/2000), Denmark (361-4), China (BJEU06-1) and South-Korea (IV3140) although this clustering is not supported by high bootstrap values. A similar tree topology was obtained using ORF5 protein sequences (data not shown). The sub-clustering of type 1 is complex and cannot always be explained by geographic isolation of the strains [8]. The sequence of 07V63 adds to the increase of genetic diversity of type 1 strains and is an example of continuous genetic drift within PRRSV [24]. A recent PRRSV study in Spain [25] demonstrated that Spanish isolates from different years show continuous evolution and increase in heterogeneity and that different genotypes and variants within the genotypes co-circulate.

Also, phylogenetic trees using Nsp2 were constructed (Figure 3B). Sequences from all known full length genotype 1 strains (Table 4) were included. Essentially, the same topology can be observed as for ORF5. A cluster of LV-like strains is evident and supported by high bootstrap values. As was already observed from the ORF5 phylogenetic tree, Amervac and SHE are very closely related as is the case for strains 01-CB1 and LV. 07V063 clusters apart from LV and is genetically distinct from the LV prototype.

## Conclusions

By using a simple random PCR cloning approach we obtained PRRSV sequence data from a recent European PRRSV isolate of unknown genetic background. This approach can be used to obtain partial genome sequences from for instance East-European type strains (for which until present, no full length genomes are available) and to get a better knowledge of the increasing PRRSV variability. We also showed that the isolate sequenced in this study is genetically different from prototype LV.

## List of abbreviations

PRRSV: porcine reproductive and respiratory syndrome virus; RT-PCR: reverse transcriptase polymerase chain reaction.

## Competing interests

The authors declare that they have no competing interests.

## Authors' contributions

JVD carried out the molecular characterization and drafting of the manuscript. MG isolated the virus and performed IPMA analysis. HJN participated in coordination and supervision of this work. PLD participated in the design and coordination of the study and drafting of the manuscript. All authors read and approved the manuscript.
